# The first mitochondrial genome of a fern sawfly, *Strongylogaster xanthocera* Stephens, 1835 (Hymenoptera: Tenthredinidae)

**DOI:** 10.1080/23802359.2021.1886019

**Published:** 2021-03-15

**Authors:** Yihan Liu, Meicai Wei, Gengyun Niu

**Affiliations:** College of Life Sciences, Jiangxi Normal University, Nanchang, China

**Keywords:** Mitochondrial genome, next-generation sequencing, phylogeny, *Strongylogaster xanthocera*

## Abstract

The first mitochondrial genome of a fern sawfly, *Strongylogaster xanthocera* Stephens, 1835 was determined. The sequence is 15,210 bp in length, including 37 typical mitochondrial genes. Four tRNA gene arrangements were observed. This mitochondrial genome provided an essential resource for addressing taxonomic issues and studying molecular evolution.

*Strongylogaster* Dahlbom, 1835 is a large genus of Selandriinae of Tenthredinidae with more than 50 known species. The systematic position of Selandriinae and the sister group of the subfamily within Tenthredinidae is uncertain to date (Wei and Nie 1998; Taeger et al. 2010). No mitochondrial genome of Selandriinae species has been reported. To promote Tenthredinidae’s phylogeny by increased sampling, we herein describe the nearly complete mitochondrial genome of *Strongylogaster xanthocera* Stephens, 1835.

Samples of *S. xanthocera* (CSCS-Hym-MC0070) were collected in Baotianman Forest Meteorological Station, Neixiang, Nanyang, Henan, China (33.49 N, 111.93 E) in 2018, and are deposited at the Asia Sawfly Museum, Nanchang (ASMN) repository (Meicai Wei, weimc@126.com) under the voucher number CSCS-Hym-MC0070. Whole genomic DNA was extracted from the thorax muscle of the specimen using the DNeasyR Blood & Tissue Kits (Qiagen, Valencia, CA). Genomic DNA was prepared in 150 bp paired-end libraries, tagged, and analyzed with the high-throughput Illumina Hiseq 4000 platform. Genomic DNA sequences were assembled using MitoZ (Meng et al. 2019); further validation was conducted in Geneious Prime version 2019.2.1 (https://www.geneious.com) using *Corymbas maculocollaris* (unpublished) as a reference. Annotations were generated in the MITOS web server (Bernt et al. 2013) and revised when necessary. Amino acid sequences were aligned by TranslatorX (Abascal et al. 2010) and concatenated with SequenceMatrix v1.7.8 (Vaidya et al. 2011). The phylogenetic tree was constructed using Bayesian inference (BI) with PhyloBayes (Lartillot et al. 2009) under the MTART + CAT models conducted on the CIPRES webserver (Miller et al. 2010).

Two contigs were generated by MitoZ. The effective sequences were 15,736 bp long, but no *trnC*, *trnI*, and *trnW* gene sequences were retrieved. By consistently obtaining similar coverage of the assembly contigs by Geneious, we were able to confirm the 247 bp region contained three tRNA genes (*trnC*, *trnI*, and *trnW*). The whole control region failed to be assembled in the above two methods, which was common for Hymenoptera, possibly due to very high AT content. Thus, the nearly complete mitochondrial genome of *S. xanthocera* was 15,210 bp in length. In total, 14,916 sequences were annotated, containing 13 protein-coding genes (PCGs), 22 transfer RNA (tRNAs), and two ribosomal RNA (rRNAs). Compared with the ancestral gene arrangement of insects (Boore 1999), *trnR* translocated to upstream of *trnN*, and the ancestral pattern of *s-rRNA*-control region-*trnI* (+)-*trnQ* (–)*-trnM* (+)-*nad2*-*trnW* (+)-*trnC* (–) clusters were rearranged to *s-rRNA*-*trnM* (–)-control region-*trnC* (–)-*trnI* (+)-*trnW* (+)-*trnQ* (–)-*nad2*, which were novel to the basal Hymenopterans.

The overall base composition was as follows: A (43.0%), T (38.6%), C (10.9%), and G (7.4%), indicating significant A + T bias. The length of the PCGs accounted for 73.7% of the mitochondrial genome. Among the 13 PCGs, *nad5*, *nad4*, *nad4L*, and *nad1* were encoded on the reverse strand (N-strand), and the rest nine were encoded on the forward strand (J-strand). Three PCGs originated from ATT, six PCGs originated from ATG, three PCGs originated from ATA. The remainder originated from ATC. All the PCGs terminated with stop codon TAA, whereas *cox1* ended with the incomplete codon T. There was two gene overlaps among *atp8–atp6* (7 bp) and *nad6-cob* (1 bp). The *rrnS* and *rrnL* genes were 853 bp and 1339 bp in length, respectively, and located between *trnL1* and *trnM*, separated by *trnV.*

For the purpose of clarifying the position of *S*. *xanthocera* within Tenthredinidae, 10 unsaturated amino acid sequences (*nad3, atp8,* and *nad4L* were excluded) of 55 Hymenopteran were used to construct the phylogenetic tree (Figure 1). The topology is similar to the previous work, with the entire Tenthredinoidea forming monophyly (e.g. Wu et al. 2020). Within the Tenthredinoidea, *S. xanthocera* is the sister group of Allantinae (Tenthredininae (Megabelesinae (Blennocampinae + Fenusinae))).

**Figure 1.**
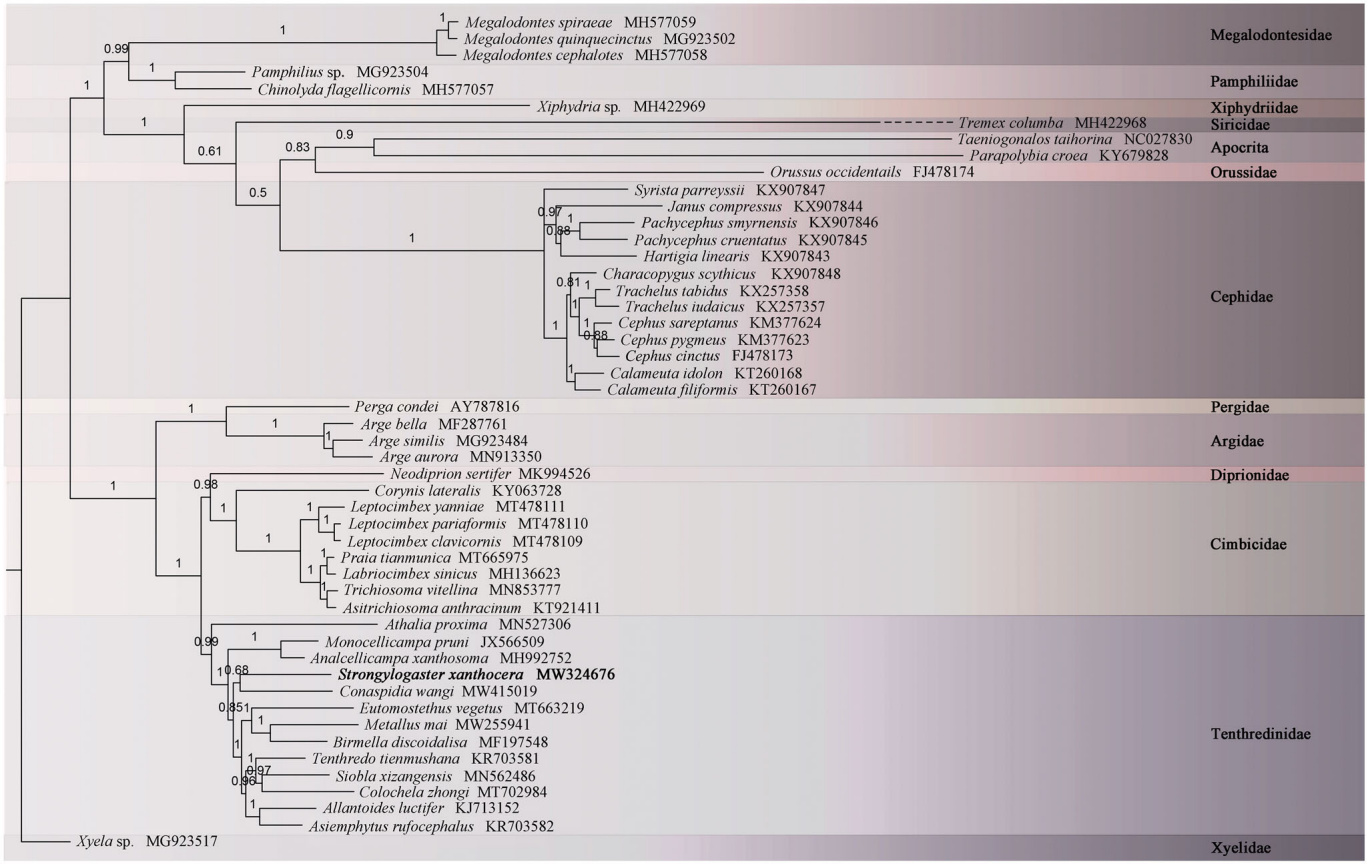
Phylobayes tree based on the combination of ten unsaturated amino acids. The numbers above each node correspond to the posterior probabilities.

## Data Availability

The genome sequence data that support the findings of this study are openly available in GenBank of NCBI at [https://www.ncbi.nlm.nih.gov] (https://www.ncbi.nlm.nih.gov/) under the accession number MW324676. The associated BioProject, SRA, and BioSample numbers are PRJNA671610, SRR13162156, and SAMN16686939 respectively.
